# Gastric pouches and the mucociliary sole: setting the stage for nervous system evolution

**DOI:** 10.1098/rstb.2015.0286

**Published:** 2015-12-19

**Authors:** Detlev Arendt, Elia Benito-Gutierrez, Thibaut Brunet, Heather Marlow

**Affiliations:** 1 European Molecular Biology Laboratory, Meyerhofstrasse 1, 69012 Heidelberg, Germany; 2 Centre for Organismal Studies, University of Heidelberg, Im Neuenheimer Feld 230, 69120 Heidelberg, Germany

**Keywords:** gastric pouches, mucociliary sole, nervous system evolution, Reissner's fibre, bHLH, fox transcription factors

## Abstract

Prerequisite for tracing nervous system evolution is understanding of the body plan, feeding behaviour and locomotion of the first animals in which neurons evolved. Here, a comprehensive scenario is presented for the diversification of cell types in early metazoans, which enhanced feeding efficiency and led to the emergence of larger animals that were able to move. Starting from cup-shaped, gastraea-like animals with outer and inner choanoflagellate-like cells, two major innovations are discussed that set the stage for nervous system evolution. First, the invention of a mucociliary sole entailed a switch from intra- to extracellular digestion and increased the concentration of nutrients flowing into the gastric cavity. In these animals, an initial nerve net may have evolved via division of labour from mechanosensory-contractile cells in the lateral body wall, enabling coordinated movement of the growing body that involved both mucociliary creeping and changes of body shape. Second, the inner surface of the animals folded into metameric series of gastric pouches, which optimized nutrient resorption and allowed larger body sizes. The concomitant acquisition of bilateral symmetry may have allowed more directed locomotion and, with more demanding coordinative tasks, triggered the evolution of specialized nervous subsystems. Animals of this organizational state would have resembled Ediacarian fossils such as *Dickinsonia* and may have been close to the cnidarian–bilaterian ancestor. In the bilaterian lineage, the mucociliary sole was used mostly for creeping, or frequently lost. One possible remnant is the enigmatic Reissner's fibre in the ventral neural tube of cephalochordates and vertebrates.

## 1. Introduction

**Figure 1.**
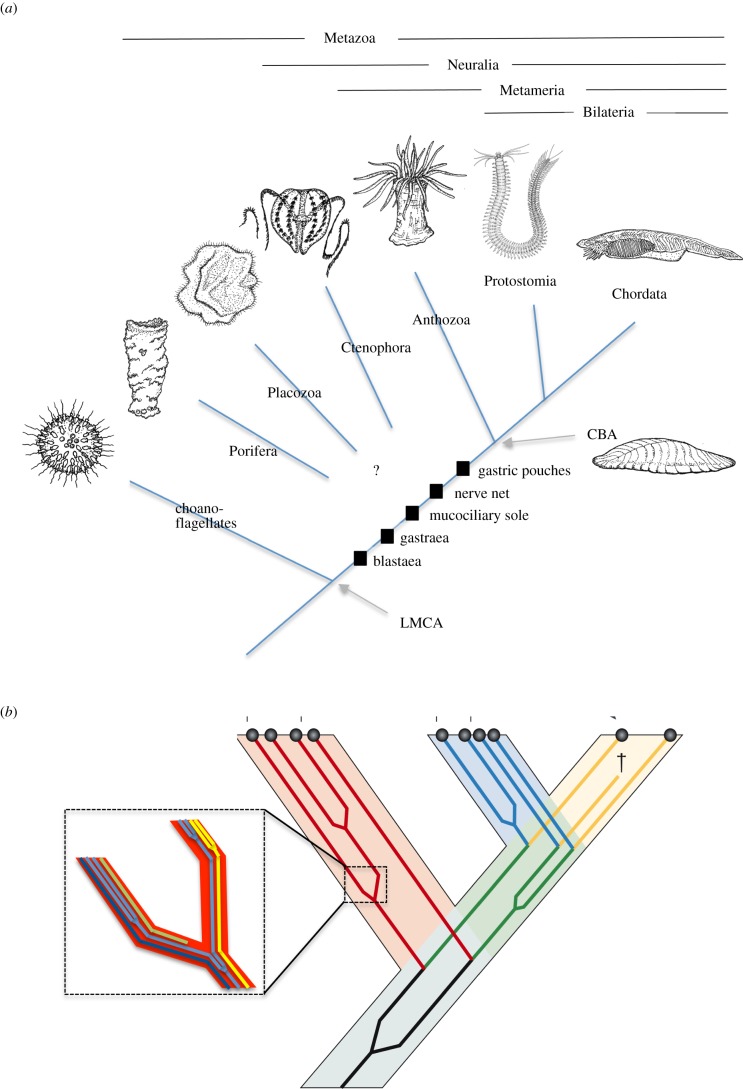
Cell-type diversification in metazoan evolution. (*a*) A simplified tree of metazoan evolution, with major inventions mapped. CBA, cnidarian–bilaterian ancestor; LMCA, last metazoan common ancestor. (*b*) The interrelationship between species, cell type and molecular evolution. The scheme shows how the cell-type tree is embedded in the species tree, and the inset shows how cell-type diversification is paralleled by subfunctionalization (yellow and dark blue lines), divergence (bright blue line) and neofunctionalization (green line) of molecules and cellular modules. It contains examples for each possible mode of cellular diversification—via functional segregation, divergence or co-option of differentiation modules. Functional segregation implies that molecular machines are differentially distributed between emergent sister cell types. At gene level, functional segregation leads to the loss of entire differentiation modules from the specializing cells. This is distinct from functional divergence, where functions (and modules) are retained and modified in different directions.

Reconstructing the evolution of the nervous system is one of the most exciting challenges of current evolutionary research. Almost certainly, a nervous system was present in the last common ancestor of cnidarians and bilaterians (cnidarian-bilaterian ancestor, CBA); yet, since the early metazoan tree remains unsolved (figure 1*a*), we cannot tell when a nervous system first emerged. Also, it is possible that the body plan of sponges or placozoans was secondarily simplified [1–3], which might have implied secondary loss of nervous system components. One way to approach the early evolution of the nervous system is to [4] track the emergence and diversification of its constituent cell types—sensory receptors, neurons and effector cells. The sister cell-type concept [5] posits that cell-type evolution can be resolved into a scheme of stepwise diversifications, giving rise to sister cell types [5]; and a recent study comparing cellular transcriptomes indicates that a hierarchical tree structure indeed dominates cell-type interrelationships [6]. This review accordingly addresses cell-type diversification in early metazoans and how it paved the way towards the birth of the first neurons. In favour of this approach, the support for a given branching order of cell-type trees is quantifiable and thus testable, meaning that the hypotheses on cell-type interrelationships put forward here can be challenged by comparative whole-organism single-cell profiling approaches [7] in the future.

Cell-type diversification involves the segregation and divergence of existing cellular modules or functions [5]. One prominent example is the division of labour that accompanied the birth of the first neurons and muscle cells (see §2). The general scheme in figure 1*b* illustrates how the evolution of cellular modules may have underlain the evolution of cell types, which in turn triggered species evolution—visualized here by encapsulated module, cell types and species trees. Following this scheme, this review provides a first survey on how, in practice, the interplay between molecular, cell type and species evolution may have shaped early metazoans, that is, it tries to track the cellular and molecular diversification events that underlay the metazoan species tree depicted in figure 1*a*. As much as it is clear that we are very far from certainty about almost all of these events, it is also true that first insights can be gained (and tested by comparative cell-type analysis, see above). A plethora of available comparative structural and ultrastructural data, recently complemented by molecular studies on basal metazoans (and on the metazoan outgroup, the choanoflagellates), allows a preliminary sketch of early metazoan cell-type evolution (that is attempted here).

The starting point will be the first metazoan, which most probably resembled a sphere of epithelialized choanoflagellate-like cells, the ‘choanoblastaea’ [8]. The recent characterization of differentiation modules in the unicellular choanoflagellates has provided rich information on the molecular composition of these cells and their multifunctional nature. The inward fold of one-half of the sphere then led to the next important ancestor, the gastraea. We will hypothesize that the simple, bilayered, cup-shaped anatomy of this ancestor was reflected by a first diversification at the cellular level, including: (i) the external ‘ectochoanocytes’, mediating interactions with the environment; (ii) the internal ‘endochoanocytes’ that specialized towards particle capture and nutrient resorption; and (iii) the ‘kopeocytes’, regulating water currents around the gastric opening. Subsequently, the kopeocytes may have further diversified into cell types specializing in ciliary movement and control, mucus production and secretion of digestive enzymes, thus enabling external digestion and forming a ‘mucociliary sole’ around the gastric opening; the endochoanocytes into cell types specializing in internal nutrient distribution and resorption; and the ectochoanocytes into cell types specializing in mechanosensation, information integration and body contractions in response to environmental stimuli. These latter cells would have constituted the first nerve net, which possibly controlled some form of amoeboid locomotion. Such equipped animals may have been close to some simple Ediacaran fossils that have been interpreted as grazing on algal mats with a mucociliary sole for external digestion [3].

Building on this, we will hypothesize that two important changes in the body plan enhanced efficiency of mucociliary sole feeding: (i) the transition to bilateral symmetry, which enabled directed movement, facilitated food search and avoided grazing the same area twice and (ii) the extensive folding of the inner surface into metameric pouches, which optimized nutrient distribution and resorption. We propose that, with these changes, basal metazoans had reached the organizational grade of the Ediacaran fossil *Dickinsonia*, which may have been close to the last common ancestor of cnidarians and bilaterians (that we collectively refer to as ‘Metameria’, figure 1*a*). With these animals, the stage was set for nervous system evolution: further diversification of the cell types making up the nerve net and the mucociliary sole gave rise to the plethora of receptor cells, neuron types and muscular and ciliary effector cells present in today's cnidarians and bilaterians.

Another comprehensive view of early metazoan body plan and nervous system evolution has recently been put forward [8]. While initial steps of metazoan evolution (such as the blastaea, see below) are in line with the scenario presented here, the CBA is subsequently derived from modified sponge larva—while the scenario presented here favours an uninterrupted sequence of benthic adults, starting with the gastraea (which each developed via swimming larval stages).

## 2. The choanoblastaea, a sphere of choanoflagellate-like cells

**Figure 2.**
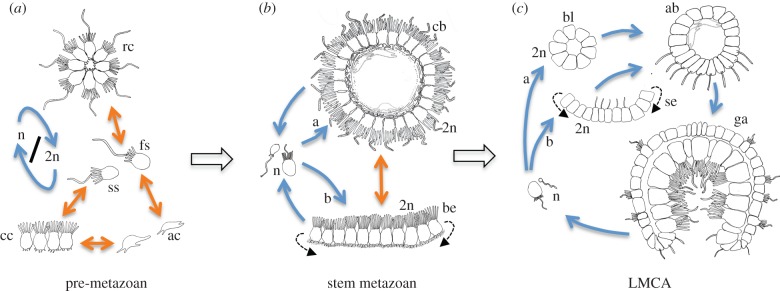
The origin of metazoans, involving a change from facultative transitions between forms (orange arrows) to fixed life-cycle transitions in development (blue arrows), and a move from temporal differentiation of cell types to spatial differentiation. Block arrows indicate direction of evolutionary change. (*a*) Pre-metazoan state with transitions between chain colonies (cc), amoeboid cells (ac), slow swimmers (ss), fast swimmers (fs) and rosette colonies (rc). 2n and n indicate life cycle transition between diploid and haploid state. (*b*) Stem metazoan. Variant (a): The zygote develops into a swimming choanoblastaea (cb). Variant (b): The zygote develops into a benthic, epithelial, biofilm-like organism (be). Transitions between the two forms might have occurred by epithelial curvature (stippled arrows) and closure. (*c*) Last metazoan common ancestor (LMCA). The zygote (2n) develops via cleavage into a blastula (bl; variant a), or, into a sheet-like embryo (se; variant b), which inverts into an amphiblastula larva (as seen in calcareous sponges; stippled arrows). The amphiblastula gastrulates into the adult gastraea (ga), which produces gametes and closes the life cycle.

Stem metazoans were likely composed of one main characteristic cell type, resembling, structurally and functionally, the choanoflagellates [8–10; see however 11]. Figure 2 sketches a possible trajectory from choanoflagellate-like pre-metazoans to the last metazoan common ancestor, involving a transition from multiple solitary and colonial forms [12,13] to a fixed life-cycle. These ancestors represented an epithelium [14] that took the shape of a sphere, called choanoblastaea [8], which later gave rise to the cup-shaped gastraea (figure 2; see also §3). They were swimming or lived partially benthic in shallow waters, filtering and concentrating dissolved organic carbon and bacteria as microphageous suspension feeders [3,15]. Building on the resemblances between choanoflagellates and sponge choanocytes, classical authors agreed that the first metazoan cells had an apical collar with an undulating flagellum [16,17] (figure 2*b*). However, a modern blastaea concept can be much more detailed on the ultrastructure of these cells, as inferred from electron microscopy and from the impressive list of metazoan proteins shared with the choanoflagellates [18]—which were necessarily present in the earliest metazoans.

### (a) The first metazoan cells: choanoflagellate-like and multifunctional

One important detail in the context of this review is the fine *glycocalyx mesh* of the microvillar collar that served as a mucoid filter [12], as well as another mesh of mucus that joined the collars of neighbouring cells [19,20]. This means that, from the very beginning, metazoan feeding was mucociliary—using both motile cilia and mucus for food capture. However, digestion was intracellular at first and took place in the same multifunctional cells that also trapped the food—just as in unicellular eukaryotes. (These two steps of feeding were spatially segregated only in more advanced metazoans, by division of labour, as will be outlined below.) Food particles were taken up by *phagocytosis* or *pinocytosis*, followed by intracellular lysosomal degradation [21]. Most probably, nutrients were shared with cells inside the choanoblastaea via the primary body cavity [8].

Other functions related to the reception and integration of, and reaction to, environmental stimuli [15]. (These were later inherited by the first neurons, as will be outlined below.) The flagellar cilia were both motile and sensory (as reported for unicellular eukaryotes and human airway cilia), and thus contained a very short and direct sensory-effector arc [22]. Chemosensation involved guanylyl cyclases signalling via cGMP [23]. Five subfamilies of transient receptor potential (TRP) channels were present [24], among which TRPV (vanilloid TRP) channels likely played an ancient role in mechanosensation, as they mediate mechanosensation in *Paramecium* and *Chlamydomonas* [25], human airway epithelia [26] and *Caenorhabditis elegans* sensory cells [27]. In *Chlamydomonas*, these channels localize to the proximal region of the cilia, where active bending is restricted (and self-activation avoided) [25]. Proteins required for action potential generation and propagation were likewise present in early metazoans, such as voltage-gated sodium [28,29], potassium and calcium channels [30]. Mechanical and chemical stimuli triggered depolarization of the ciliary membrane and calcium influx into the cilium that had a direct effect on ciliary beating such as reversal or inhibition, as reported for unicellular eukaryotes, cnidarians, ctenophores and bilaterians [31]. Adding to this, choanoflagellates and metazoans share synaptic adhesion/signalling molecules, a primordial neurosecretory apparatus active on the apical cell surface (but no presynaptic active zone proteins) [32], postsynaptic density proteins such as Homer or Shank [33,34] and a variety of receptors including the metabotropic glutamate receptor [35] (but not ionotropic glutamate receptors, which only appeared in the metazoan stem). Many of these molecules and associated functions were key to the evolution of the first neural cell types, as will be detailed below.

On the effector side, early metazoan cells already possessed a complex machinery for cellular contractions and shape changes based on a dynamic actomyosin cytoskeleton. Actin-based movement involved a slow and a fast version of myosin II that responded in a different manner and with different velocities to Ca^2+^ signalling [36]. The slow non-muscle myosin probably played a role in various cellular processes involving contraction, such as integrin-mediated basal adhesion, cell–cell adhesion dynamics via adherens junctions or apical constriction [37]—processes that were key for the evolution of metazoan epithelia. The slow myosin is also involved in amoeboid movement [38], which probably belonged to the behavioural repertoire of early metazoan cells. In addition, we can infer that early metazoan cells formed lamellipodia and filopodia, using proteins that reorganize cortical actin filaments such as the Arp2/3 complex, the actin cross-linking protein fascin, and myosin X, a myosin with pleckstrin homology domains that associates with regions of dynamic actin [39,40]. Filopodia are highly dynamic structures that probably played a role in anchoring and stabilizing cells in the blastaea (figure 2*a*,*b*).

## 3. The microphageous gastraea

**Figure 3.**
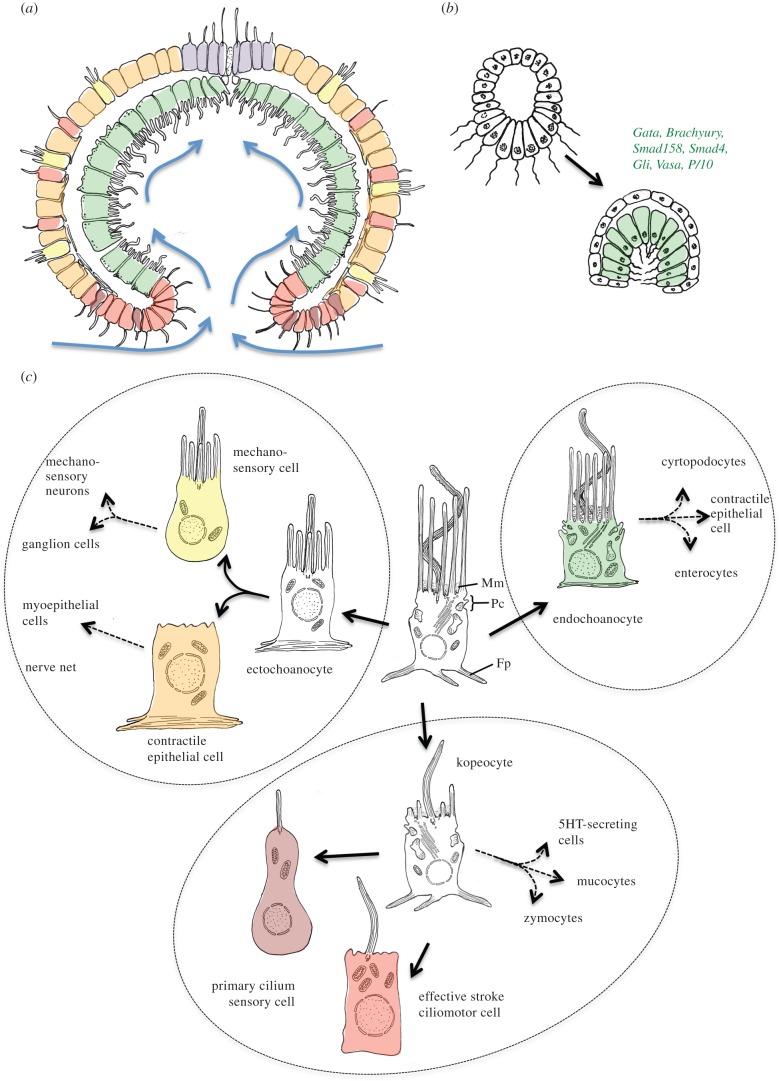
The gastraea, the last common ancestor of living phyla. (*a*) The gastraea. This cup-shaped animal resulted from simple infolding of the initial blastaea and first cell-type diversification. Colours relate to similar cell types in (*b*) and (*c*). (*b*) Infolding of the amphiblastula larva of calcareous sponges into a cup-shaped transitory stage that resembles a gastrula. The inner cells express endomesodermal markers, after [41,42] and various sources. (*c*) The initial diversification of metazoan cell types into sensory-contractile cell types and cell types related to feeding. The colour scheme matches the cells of the gastraea in (*a*). A multifunctional choanocyte-like cell type gives rise to ectochoanocytes, endochoanocytes and kopeocytes (black arrows). These ancestral cells had an apical, undulating cilium or flagellum, propelling water away from the cell, surrounded by a circle of long, contractile, actin-containing microvilli. A well-developed Golgi complex produces lysosomes for intracellular digestion, and a prominent endocytotic machinery mediates phagocytosis (Pc). Mm, mucoid mesh; Fp, filopodia.

It is a long-standing notion that the second step in the evolution of the metazoan body was the transformation of the spherical blastaea into the gastraea [8,9], which may have been close to the last metazoan common ancestor (figure 1*b*). The term ‘gastraea’ means ‘animal with a primitive gut’ and was coined by Ernst Haeckel [9]. Its most characteristic feature is the infolding of the lower body surface, resulting in an outer ectoderm, an inner gastroderm and a gastric opening (figure 3*a*). From Haeckel's times until today, strong support for the gastraea hypothesis comes from the prevalence of gastrula-like stages during animal development that are interpreted as recapitulation of the gastraea-like ancestral body plan. Even in sponges, a transitory cup-like stage forms during metamorphosis, as recently confirmed [41]; and in cnidarians and ctenophores, the overall body form resembles that of a gastrula during the entire life cycle—at larval, polyp and medusa stages. The ancient gastraea is originally assumed as creeping on the sea floor, with the gastric opening facing downwards (while other authors consider the gastraea a predominantly planktonic organism [8]): plausibly, this would have closed off the gastric cavity from the environment and thus allowed higher nutrient concentrations. In line with this, some sponge larvae settle with the blastopore facing downwards [41]. Sponges might be regarded as representatives of a gastraea-type organization (which then would have undergone surface extension independent of other metazoan lineages; however, it cannot be excluded that, alternatively, sponges lost ancient features [1,2]).

Modern support for the classical gastraea hypothesis comes from molecular data. A larger set of genes specifying endomesoderm has recently been found expressed in the ciliated micromeres of the amphiblastula larva of the calcareous sponge *Sycon ciliatum*, which later invaginate and transdifferentiate into the choanocytes of the adult sponge [42] (figure 3*b*). This supports homology of sponge inner layer with the endomesoderm (i.e. the gastroderm) of other animals. Additional support comes from the conserved expression of conserved transcription factors such as *brachyury*, and signalling molecules such as *hedgehog*, in the blastoporal tissue of various metazoans [43,44].

Given the structural and functional resemblance between sponge choanocytes and choanoflagellates, it seems obvious that the gastraea, just like the blastaea, used choanocyte-like cells to feed on dissolved organic carbon or microbes suspended in the inflowing seawater (microphagy [3]). However, it is also plausible that these cells had already started to diversify, with inner cells specializing more on feeding functions and outer cells on functions important for the interaction with the environment—such as protection, different sensory modalities and primitive body movements. For these outer versus inner cell types, we propose the names ‘ectochoanocytes’ and ‘endochoanocytes’, reflecting their choanocyte-like appearance and their developmental origin from ecto- and endoderm, respectively (figure 3*c*). Another founder cell type that will be key to the scenario developed here is hypothesized to have existed at the interface between ecto- and endochoanocytes, around the gastric opening (the ‘kopeocytes’; see §3c below). These cells specialized in the generation and control of water currents (and later gave rise to the cell types constituting the mucociliary sole; see §4).

### (a) Endochoanocytes: cell types specialized in feeding

Besides the functions related to feeding, such as flagellar pumping, microvillar-mucoid filtering and pino- and phagocytosis [12,45], the endochoanocytes likely also retained some gustatory chemosensory functions, as recently proposed [46], together with the capacity for cellular contraction—mostly via the ‘slow’ myosin II, which was likely present in the ancient gastroderm as it is broadly expressed in sponge choanocytes and in the cnidarian gastroderm [36].

### (b) Ectochoanocytes: sensory-contractile cell types

Ectodermal mechanosensory cells with obvious resemblance to sponge choanocytes have frequently fuelled cell-type comparisons across metazoans [47–49]. For example, the structural resemblance of the hair cells of the ear has long been noted [8,46,50,51]. Just like choanocytes, these cells bear a central cilium, called ‘kinocilium’, surrounded by or adjacent to interconnected microvilli, called ‘stereocilia’ (figure 3*c*). Besides the vertebrate acustico-lateralis system, similar mechanoreceptors are found in annelids [52] or acoels [53], and in great diversity in cnidarians: in cerianthids, sea anemones, corals and medusozoans [49,54–56]. Remarkably, the mechano-‘sensory’ cilium of these cells appears to derive from a motile cilium and sometimes retains residual motility (e.g. in the insect chordotonal neurons [57] or in hair cells [58]); the axoneme of the kinocilium accordingly possesses a central doublet of microtubules as otherwise characteristic for motile cilia. Interestingly, mechanosensory cells such as vertebrate hair cells and insect chordotonal cells express TRPV receptors [57], which might be inherited from early metazoans (see §2a). In line with an ancient role of these receptors in mechanotransduction, a mammalian TRPV receptor rescues the mechanosensory (and osmosensory) properties of a TRPV channel in nematode worms [59]. Notably, these cells have not (yet) been described for sponges—as they occur in the ectoderm of virtually all other phyla, however, they were very likely present in the ancient gastraea, too.

Contractile ectodermal fibres are found in sponges [60], cnidarians [61,62], ctenophores [63] and in many bilaterians, making it likely that they were also present in the ectoderm of the gastraea (figure 3*c*). Sponge contractile movements are largely mediated by the slow ‘non-muscle’ myosin, which is expressed in most cell types in *Tethya wilhelma* including the contractile pinacocytes [36,60]. Ectodermal contraction appears to have always involved intercellular communication by glutamate and GABA: in sponges, contraction waves involve paracrine intercellular signalling, with glutamate triggering and GABA inhibiting contraction [64,65]. Intercellular propagation of the wave is slow and involves the metabotropic glutamate receptor [15].

As will be explained in more detail below, the segregation of sensory-integrative mechanosensory cells versus contractile epidermal cells (figure 3*c*) may have represented the first step towards the evolution of the later nerve net. This is in line with the classical idea that mechanosensory-contractile cells gave rise to the first neural circuit by division of labour [5], as proposed by Kleinenberg [66] or Mackie [67]; and with more recent suggestions that the sponge contraction waves can be regarded ‘forerunners’ of nervous activities [15,60].

### (c) Kopeocytes: cell types producing and sensing directional water flow

A key characteristic of the gastraea would have been a flow of water into the gastric opening, to ensure fresh supply of suspended particles and to maximize feeding. In contrast with the movement away from the epithelial surface generated by a standard flagellum, this new flow was needed in parallel to the epithelial plane. Therefore, and inevitably, the motor cilia generating this current and the cells bearing these cilia must have shown planar polarity [68] and an oar-like motion (effective stroke) [69], similar to the motile cilia of swimming larvae. (Note that flagella and effective stroke cilia differ structurally [70].) It appears plausible that such cells, shoveling water and suspended particles into the gastric cavity with effective stroke cilia, were first situated around the gastropore, red and brown in figure 3*c*. We propose to refer to these cells collectively as *kopeocytes* (from greek ‘kopeon’, the oar). Besides the effective stroke cilium, the kopeocytes presumably retained the apical secretory apparatus and basal contractility. By division of labour, such cells may have generated a number of important sister cell-type descendants, such as effective stroke ciliomotor cells, primary cilia sensory cells, specialized contractile cells and the various cell types of the mucociliary sole, as will be further outlined below.

In sponges, pigmented ring cells and apopylar cells may represent direct kopeocyte descendants. The apopylar cells, regulating water outflow from the choanocyte chambers [20], are contractile and express the fast type of ‘myosin II’ [36]; and ‘fast’ myosin II is also expressed around the pigmented ring in *Amphimedon* larvae [36], which also bears effective stroke cilia [71]. In other animals, the occurrence of effective stroke cilia often relates to the production of water currents through openings that may ultimately relate back to the gastropore, suggesting that the respective cells are kopeocyte descendants. However, it is not yet clear whether effective stroke ciliated cells represent a uniform cell type or, alternatively, evolved independently one or even many times. Two conserved transcription factor families have been identified that control the motile ciliogenic program in all metazoans: the forkhead-related FoxJ [72] and Rfx, a winged helix factor [73–75]. However, the FoxJ/Rfx network appears to specify effective stroke and flagellar motile cilia alike [76] and thus cannot solve this question.

Sensory cells with primary cilia, such as the mammalian kidney epithelia, are likewise widespread in animals and have recently been found in sponges—inside of the outflow opening, the osculum, which senses chemical signals and water turbulences and initiates contractile responses [15,60,65,77] (see below). This indicates that they may be part of the cellular repertoire situated around the gastric opening and may have arisen from kopeocytes by sensory-motor division of labour (figure 3*c*). Lacking the central pair of microtubules and the spoke apparatus (as well as the actin collar), primary cilia are non-motile. They sense flow (vibration) via TRP channels [78], responding to a deflection of the cilium owing to water current with a calcium signal. In vertebrates, the two TRPP duplicates PC1 and PC2 comprise a rheosensory complex that translates deflection of the primary cilium into signals [78]. Sponge primary cilia also appear to employ a TRPP (PC2) channel, as indicated by sensitivity to three pharmacological inhibitors blocking this channel in vertebrates [77]. Interestingly, primary cilia also respond to chemicals, and given that both rheosensation and effective chemosensation are necessarily linked to planar flow of water (and/or mucus, see below), it is plausible that primary cilia may relate back to the proposed diversification of kopeocytes (figure 3*c*). However, because the major characteristic of primary cilia is loss—of the actin collar and of the central microtubule doublet and the spokes—it is also possible that they evolved several times independently (e.g. in sponges versus other animals). To find out, it will be interesting to determine whether a common cellular module exists that is not found elsewhere and positively defines all primary cilia, or subsets of them, across animals.

### (d) Diversification of mechano- and chemosensors: the ATH and ASC bHLH superfamilies

While the distinct presence of mechanosensors (with actin collar and kinocilium) and of presumed rheo- or chemosensors has been postulated based on the frequent parallel occurrence of both sensory types in basically all metazoans, this notion finds support from the molecular evolution of the class A superfamily of bHLH of transcription factors. While this class is exclusive to metazoans [79], it already comprised three members—an achaete-scute (ASC), an atonal (ATH) and an E12/E47 superfamily member [80]—before the sponges branched off the metazoan lineage. Two of these—the achaete-scute and atonal families—are strongly associated with the specification of distinct sets of sensory cells across metazoans [57].

The atonal family genes specify mechano- and photoreceptor cells in a broad range of animals [57,81], such as the hair cells [82], Merkel cells [83,84] and ganglion cells in vertebrates, the chordotonal stretch and auditory receptor cells [57] and ommatidial photoreceptor cells [85] in insects and, as extrapolated from gene expression, the mechanosensory and photoreceptive cells in jellyfish [86]. (Note that the *atonal*-dependent rhabdomeric photoreceptor cells couple opsin-based phototransduction to mechanosensitive TRP channels [87].) An ancestral role of atonal in sensory cell specification is supported by mutual rescue of atonal function between *Drosophila* and mouse [88,89]. Atonal family members have been reported to directly control a gene regulatory network implementing mechanoreceptor morphology. In the *Drosophila* chordotonal mechanoreceptors, *atonal* activates Rfx and Fd3F (a Foxj orthologue [90]) that synergize to implement cellular modules characteristic for the mechanosensory cilium. This includes modules required for motility and the TRPV channels [91]. We can thus infer that *atonal* genes probably played an early role in ectochoanocyte specification. In line with this, the atonal superfamily member *bHLH1* appears to be involved in the specification of flask-shaped sensory cells in the demosponge *Amphimedon*.

By contrast, the achaete-scute-like bHLH factors have been frequently implicated in the specification of chemosensory neurons. In the *Drosophila* chemosensory system, ASC is required for the specification of gustatory receptor cells in larvae [92] and in adults [93]. In vertebrates, ascl proteins are required for the specification of Merkel-like, 5HT+ taste bud cells [94] and for the 5HT+ sour taste type III taste bud receptors in mice [95,96]. Also, mouse Mash1 has been implicated in the control of catecholaminergic, chromaffin cell differentiation [97]. During early postnatal life in mouse and rodents, these cells possess ‘direct’ hypoxia- and CO_2_/H+-chemosensing mechanisms, and acute hypoxia causes depletion of adrenal catecholamines via a ‘non-neurogenic’ mechanism [98]. Remarkably, sensory ectodermal cells specifically express an *achaete-scute homolog* (*ASH*) gene in *Hydra* [99] and, most probably, *Nematostella* [100], consistent with the notion that achaete-scute family members play an ancient role in the specification of at least some kind of sensory neurons [101]. These findings indicate that the distinct genetic control of major sensory modalities was already in place in the last common metazoan ancestor (and thus predated the evolution of the nervous system).

Interestingly, the E2A proteins (E12/E47) have been shown to specifically bind the E-box-containing region of the smooth muscle actin promoter, regulating a smooth muscle cell differentiation marker *in vivo* [102]. Just like sponges, *Trichoplax* has an E12/E47 orthologue, indicating that the conserved function of this ancient class A bHLH protein was in the specification of cells with smooth muscle fibres (see above).

## 4. The benthic gastraea: external digestion with a mucociliary sole

**Figure 4.**
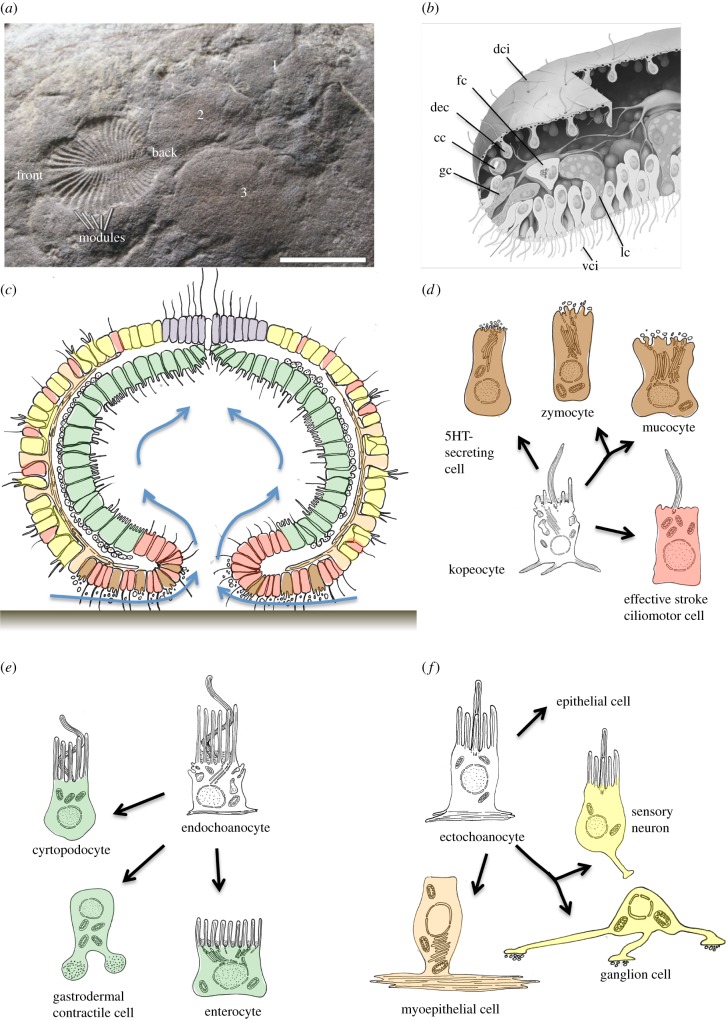
Evolution of the mucociliary sole. (*a*) Body fossil of *Dickinsonia costata* associated with a series of feeding traces, reproduced with permission from [3]. Numbers delineate the order of their formation in relation to the body fossil at the end of the series of traces (trace #3 made last). Scale bar, 2 cm. (*b*) Cell types of the placozoan *Trochoplax,* reproduced with permission from [103]. The thick ventral body surface is composed of ventral epithelial cells (vci), each bearing a cilium and multiple microvilli; lipophil cells (lc); and gland cells (gc), distinguished by their contents of secretory granules and prevalence near the margin. Dorsal epithelial cells (dec) constitute the dorsal surface. In between the dorsal epithelium and ventral plate are fibre cells (fc) with branching processes that contact each of the other cell types. cc, crystal cell; dci, dorsal ciliated cell; gc, gland cell. (*c*) A hypothetical gastraea-like ancestor with mucociliary sole. In these animals, mucocytes, zymocytes and serotonergic cells (brown) and motile ciliary cells (red) are located around the gastric opening. Contractile cells with ring-shaped fibres, excretory cells and enterocytes (all green) populate the gastric cavity. Mechanosensory cells (yellow) and contractile epithelial cells (orange) are hypothesised for a primordial nerve net. Blue arrows indicate direction of water flow across the mucociliary sole and into the gastric cavity. (*d*) Hypothetical diversification scheme for mucociliary cell types. (*e*) Hypothetical diversification scheme for endochoanocytes. (*f*) Hypothetical diversification scheme for ectochoanocytes into nerve net cells.

Various lines of evidence indicate that a new feeding mode evolved in the Precambrian ocean, referred to as mucoid-ciliary particle feeding [3] (figure 4*a*,*b*). This mode replaced the microphagy of individual cells. Food was predigested externally in a layer of mucus and the predigested nutrients transported into the body cavity with water currents. While the ancient choanocytes probably already used an intercellular mucoid mesh for particle trapping (see above), this mesh now covered the entire epithelium around the gastric opening, facing the substrate. Digestive enzymes were secreted into this mucus layer for external digestion. The coordinated and directional beating of effective stroke cilia distributed and transported the mucus over the digestive epithelium and washed the nutrients into the gastric cavity. Such hypothetical animal, which may have been close to the Eumetazoan ancestor, is depicted in figure 4*c*.

### (a) Grazing algal mats with mucus and digestive gland cells

A rich body of fossil and comparative evidence indicates that mucoid-ciliary particle feeding evolved in early metazoans. Feeding traces indicate that one of the most characteristic fossils of the Ediacaran fauna, *Dickinsonia*, was grazing on the sea floor, which was covered by ubiquitous microbial mats [3] (figure 4*a*). It is assumed that their entire ventral body surface was a digestive ‘sole’ covered with mucus and cilia [3]. We refer to this new tissue as ‘mucociliary sole’. A digestive ventral sole, used for external digestion and movements, also exists in today's placozoans [104] (figure 4*b*). Placozoans move by ciliary gliding with the thick mucociliary sole facing the sea floor, and by concomitant contraction of the syncytial fibre cells (figure 4*b*). Capturing of microparticles with the help of cilia and mucus persists in some cnidarians; and mucoid-ciliary food transport is also found in macrophageous cnidarians and ctenophores. For example, in the sea anemone *Calliactis parasitica* non-neuronal control of ciliary beating involves a direct chemosensory response of pharyngeal epithelial cells [105]. Ctenophores possess an elongated, ciliated pharynx with numerous gland cells secreting digestive enzymes [106]. As in cnidarians, the control of ciliary beating for food transport is non-neuronal. Nutrients are transported into a complex system of gastrovascular canals for nutrient distribution. Residual water is expelled to the outside via anal pores. Excitingly, the pharynx can be everted and used as a mucociliary sole in benthic ctenophores [62]. Finally, mucociliary epithelia are frequently found in foregut and neural midline tissue in bilaterians (that can be derived from tissue surrounding the gastric opening, see below). This includes the dorsal ciliary folds in the annelid foregut [107], the buccal field in the rotifer *Dicranophorus* [108], the endostyle/thyroid in the chordate foregut, the neurotrochoid along the neural midline in various invertebrates [109,110] and the enigmantic Reissner's fibre in chordates (see below).

### (b) Cell types of the mucociliary sole

The invention of the mucociliary sole was likely paralleled by cell-type diversification (figure 4*d*), specializing in the production of mucus, the secretion of digestive enzymes, the transport of mucus and the uptake of dissolved nutrients [45]. Mucus is largely made up of large glycoproteins called mucins [111]. Gel-forming mucins, Otogelin and von Willebrand factor are evolutionary related [111], and proteins with properties of the gel-forming mucins were identified also in the starlet sea anemone *Nematostella vectensis* [111]. Furthermore, mucus-secreting cells (‘mucocytes’, or ‘goblet cells’) are common to most animal taxa. The secretory vesicles of mucocytes are of low electron density, in contrast to those of the zymogenic gland cells (‘zymocytes’, figure 4*d***)**, which secrete digestive enzymes. The latter are found in the bilaterian foregut, in the ctenophore and cnidarian pharynx (with associated filaments) and around the digestive ventral sole of the placozoans (figure 4*b*) [45]. The zymocytes have apical microvilli and/or cilia in some groups [45], reminiscent of their choanocyte origin. Within the mucociliary sole, the motile ciliary cells specialize in moving the secreted mucus along the epithelial surface.

### (c) A link between the mucociliary cell types and *Fox* family evolution

At the molecular level, the evolutionary divergence of mucociliary cell types in metazoans appears to be closely linked to the diversification of the *Fox* gene family. Most prominently, *foxj* has been identified as an ancient key regulator of motile cilia biogenesis [72,112,113], conserved in cnidarians [72] and even sponges [114]. *Foxj* is active wherever motile cilia are present; for example, in *Xenopus*, *foxj* genes are expressed in otic vesicle, pronephros, gill structures, notochord, floorplate and ventral neural tube [115]. Another member of the *Fox* family, *foxi,* specifies the osmoregulatory ionocytes, another characteristic cell type of vertebrate mucociliary epithelia [116]; moreover, *foxA* has been found to specify small 5HT-secreting cells in the mucociliary epithelium that regulate ciliary beating [117,118]. Additional members of the *Fox* family have been implicated in the specification of mucociliary cell types: the thyroid-related transcription factors *TTF-2/foxE4*, *foxq1* and *foxA* are jointly expressed in the amphioxus and ascidian endostyle, a presumed homolog of the thyroidea that harbours mucus-secreting and motile ciliated cells; and this function of the *Fox* genes is considered ancestral for the chordates [119]. Interestingly, the metazoan *foxA, B, C, D* and *E* subfamilies are evolutionarily related [120–122] and resulted from duplications of an ancient precursor gene; in *Nematostella*, *foxB* [122] and *foxA* [123] are expressed in the ectodermal ciliated pharynx and *foxC* [122] in pharyngeal endoderm; in *Hydra*, *foxA/budhead* [124] is likewise expressed in the endoderm around the oral opening, suggesting that the mucociliary epithelium around the gastric opening represents the ancient site of activity of the *foxA, B, C, D, E* superfamily.

### (d) Diversification of endochoanocytes: enterocytes and excretory cells

With the invention of the mucociliary sole around the gastric opening, the endochoanocytes were no longer needed for mucoid particle trapping. In consequence, they diversified into more specialized cell types—enterocytes, excretory cells and contractile cells (figure 4*e*). Enterocytes are optimized towards the uptake of nutrients; either directly from the mucus or from the nutrient-enriched water of the gastric cavity. Morphologically, they still resemble the endochoanocytes in that they often bear motile cilia in combination with a collar of microvilli, at least in most invertebrates [45]. Microvilli increase the membrane surface facing the gut lumen and thus facilitate nutrient uptake. Finally, excretory cells are specialized in removing the nutrient-deprived, nitrogen-enriched water from the body cavity. While in sponges the choanocytes engender fluid motion and water removal, specialized cells exert this function in many other animals, such as the protonephridial flame cells. A choanocyte-origin has been previously postulated for enterocytes [45] and for flame cells [46], based on morphological, functional and molecular resemblance.

## 5. The benthic gastraea: evolution of the nerve net

**Figure 5.**
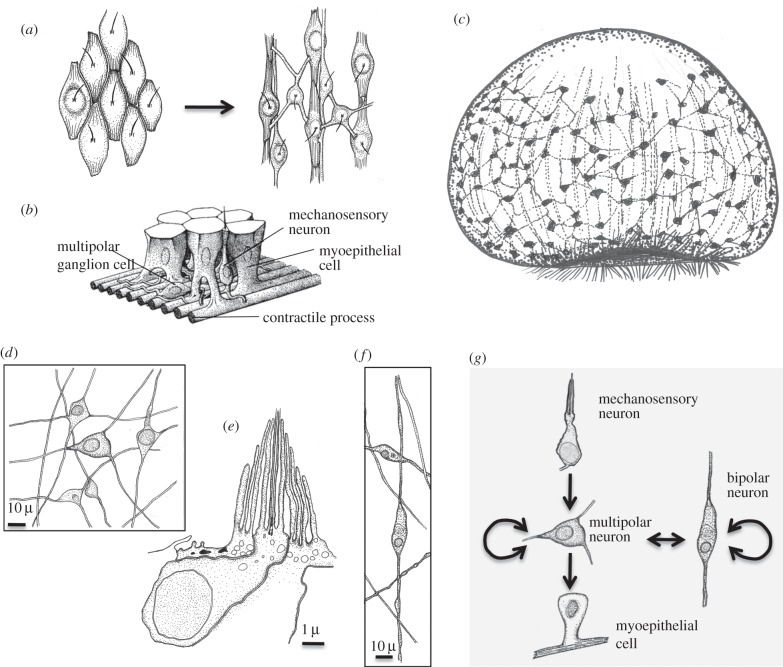
Evolution of the nerve net. (*a*) Evolution of a sensory-contractile network of neurons and muscle cells by division of labour [5,6,125], following an initial scenario proposed by Mackie [126]. Starting point is a network of multifunctional, sensory-contractile cells in the gastraea ectoderm. Contractile fibres are oriented along the apical-blastoporal body axis. Individual sensory-motor neurons innervate many muscle cells. (*b*) Cell types of the cnidarian nerve net, modified from [127]. (*c*) The neuromuscular orthogon. Muscle cells have congregated into muscle strands. The evolution of true sensory, inter- and motorneurons enables the differential and antagonistic contraction of entire muscle strands as a response to remote stimuli. (*d*) Ganglion cells in the ectodermal nerve net in *Cerianthus*, after [128]. (*e*) Mechanosensory ciliary-cone receptors in the ectodermal nerve net in *Cerianthus*, after [56]. (*f*) Bipolar cells in the ectodermal nerve net in *Cerianthus*, after [128]. (*g*) Connections and signaling directions (arrows) in the nerve net of *Cerianthus*, after [128].

Having reasoned that a benthic, gastraea-like ancestor with a mucociliary sole populated the Proterozoic sea floor, the stage is set for the evolution of the first nervous system. We will elaborate here that the first neurons emerged in this ancestor in the context of an ectodermal nerve net (figures 4*f* and 5). The initial function of this nerve net might have been the control of amoeboid locomotion of the gastraea, grazing on algal mats (resembling the movement of an amoeba, but at multicellular scale).

Nerve net-like arrangements of neurons cover the almost entire ectoderm in cnidarians and ctenophores [61,129,130–136]. In ctenophores, the ectodermal nerve net is a polygonal mesh of neurite bundles; another nerve net spans the mesoglea between the ectoderm and the endomesoderm [129]. In the cerianthids, a basal cnidarian lineage, the nerve net appears to be of purely ectodermal nature [128], which suggests that the evolution of inner layer neurons occurred independently in ctenophore, cnidarian and bilaterian lineages [136]. As sponges and placozoans do not form a nerve net, the most parsimonious assumption appears to be that a nerve net existed in the last common ancestor of cnidarians, ctenophores and bilaterians (a grouping referred to as Neuralia by Nielsen [8]); yet, in light of the current uncertainties about the placement of the ctenophores [137], the existence of the ‘Neuralia’ at the exclusion of sponges and placozoans remains unsettled: cf. figure 1*a*).

### (a) The neuromuscular orthogon

The presence of a well-developed nerve net correlates with the presence of basiepithelial muscle fibres directly innervated by nerve net neurons, in cnidarians and in ctenophores [61,67,128,132,133,135,136,138–142]. Therefore, the evolutionary origin of the nerve net appears to be closely linked to that of the musculature [44]. As a rule, the ectodermal muscle fibres associated with the nerve are oriented longitudinally, that is in parallel to the primary body axis (while the inner layer forms circular fibres [61,62]). This matches the situation in the basal cnidarian cerianthids [61], as well as the medusozoan ground pattern [62], and longitudinal muscle fibres are the first to develop underneath the epidermis in the ctenophore *Mnemiopsis* [143], followed later by circular fibres [129]. It is thus plausible that the benthic gastraea evolved longitudinal muscle fibres innervated by an ectodermal nerve net, by division of labour as illustrated in figures 4*f* and 5*a*,*b*. The perpendicular arrangement of myofibres innervated by an ectodermal nerve net is referred to as ‘neuromuscular orthogon’ ([44]; figure 5*c*). Importantly, neuromuscular orthogon and mucociliary sole appear to have overlapped, because longitudinal muscles reach the gastric opening (at least in cnidarians).

Such an ectodermal nerve net essentially controlled body wall contractions, possibly mediating local to more widespread ‘avoidance’ responses to mechanical stimuli [141]. Most probably, the nerve net first thus coordinated some very primitive form of amoeboid locomotion, which required alternating and antagonistic contraction of perpendicular myofibres, in order to elongate the body towards the desired direction and/or to change body shape in reaction to mechanical stimuli that were met on the locomotor trajectory. In essence, we hypothesize that such locomotion required more sensory integration and coordination with increasing body size (and that the increase in body size in turn resulted from the invention of the mucociliary sole with external digestion). It will be interesting to work out how an animal of this organizational grade may relate to beginnings of the ctenophore line of evolution [144], and whether placozoans pre- or postdated the invention of the nerve net (that is, whether they are ancestrally simple or secondarily miniaturized [3], having potentially lost or modified their gastric cavity, neurons and myocytes [137]).

### (b) Cell types of the nerve net

What were the cell types that constituted the first nerve net? Core units of the basal metazoan nerve nets appear to be local two- to three-cell sensory-effector reflex arcs [138] that directly innervate the effector cells (myoepithelial cells or cnidocytes in cnidarians [138], muscle cells and ciliated cells in ctenophores [129]). These arcs are interconnected at the level of the sensory neurons or ganglion cells, so that the whole epithelium functions as a single tissue-type reflex arc [145] that triggers contraction of myoepithelial cells (and/or local discharge of cnidocytes in cnidarians). Chemo- and photosensation as well as neuropeptide and indolamine release may have modulated nerve net activity.

The cellular composition of the nerve net has been studied by light- and electron microscopy as well as immunocytochemistry in numerous anthozoans [61,128,132,146,147], hydrozoans [142,148,149] and ctenophores [129,134,150] and proved rather homogenous and similar, which allows ancestral state reconstruction. Mechanosensory ‘ciliary-cone cells’ (figure 5*e*) occupy the ectodermal surface of cerianthids and sea anemone [56] (and similar cells exist in the nerve net of hydrozoan polyps [149,151,152] and ctenophores [150]). An apical collar of microvilli surrounds a single cilium [49,153], identifying these cells as ectochoanocyte descendants (see above). Many of the sensory cells form synapses [56,138], thus representing true sensory neurons. In addition, two types of ganglion cells are commonly observed, in cerianthids [128], sea anemone [132], hydrozoans [148] and ctenophores [129,134]. The small multipolar neurons (figure 5*d*) make up a large part of the nerve net in sea anemone, projecting few, thin neurites in different directions [130]. In cerianthids, these are the only neurons to directly receive sensory innervation, to connect to each other, and to directly innervate, via multiple collaterals, the underlying myofibres [128]; they can thus be regarded as the core of the ancestral nerve net [130]. By contrast, the large bipolar cells (figure 5*f*) send out neurites that are much thicker, less frequent and follow the muscle fibres [130]; they represent second order interneurons transmitting nervous excitation over longer distances, mediating for example whole body retractions [128,130]. The information flow in a prototype nerve net is exemplified for cerianthids in figure 5*g*.

### (c) The birth of first neurons by division of labour

The prevalence of mechanosensory neurons with actin collar and cilium, multipolar ganglion cells and myoepithelial cells in extant nerve nets supports the idea that the first neurons arose by division of labour from mechanosensory-contractile ectochoanocytes, as hypothesized in the diversification scheme in figure 4*f*. Similar ideas have been put forward repeatedly [5,66,67] and imply that the first synapse arose from the aggregation of vesicle-related intercellular signalling modules, including synaptotagmin, SNARE-complex components and postsynaptic receptor complexes, as extensively reviewed elsewhere [15,32,34,46,60]. This aggregation process might have occurred around adherens junctions, as suggested by the shared presence of both N- and E-cadherin in adherens and synaptic junctions [154]. In line with a division of labour scenario, cnidarian sensory neurons and ganglion cells are related to the extent that they cannot be clearly separated into two distinct cell types [139]. To test these hypotheses further, the molecular profiling of cell types constituting the nerve net in cnidarians and ctenophores will be especially rewarding—and also solve the yet unsettled question of nervous system homology between ctenophores and other neuralians [155].

### (d) Glutamate and GABA

In line with the postulated origin of nerve net neurons from ectochoanocytes, nerve net synaptic transmission appears to be largely glutamatergic, as recently postulated for ctenophores [156] and as indicated by many lines of circumstantial evidence in cnidarians. In sea anemone, NMDAR 1 immunoreactivity localizes to both nerve and epitheliomuscular cells [157] and glutamate immunoreactivity has been detected in distinct cells of the ectodermal layer and in the nerve plexus, but was absent from mesoglea and endoderm. Immunoreactivity of the nerve plexus forms a continuous ring on top of the epitheliomuscular cell layer in tentacle cross-sections [158]. Electron optics reveal the presence of glutamate in individual neurites within the nerve plexus, and in large dense-core vesicles within putative neural processes (but evidence for glutamate in synapses is yet lacking [158]). Glutamate exerts inhibitory activity on circular muscle contraction of the sea anemone *Actinia equina* [159], indicating that glutamate exhibits opposite effects on ectodermal and gastrodermal musculature. Adding to this, numerous studies report paracrine release of structurally related, amidated neuropeptides of the RFamide and other families from cnidarian nerve net neurons [136,139,157,160].

A smaller subset of cnidarian nerve net neurons has been reported GABA immunopositive [146]. In sea anemone [158]*,* GABA and GAD immunoreactivity is prominent in the outer and inner sublayers of ectoderm and endoderm, but not in the nerve plexus. In the basal ectoderm, the epithelio-muscular layer displayed an intense label, but the nerve plexus remained scarcely stained. In the outer ectoderm, GABA labelled some types of sensory neurons [158]. Just like cnidarians, ctenophores use glutamate in nerve net signalling, while GABA has been detected in muscle cells [15,156]. These data suggest that glutamate was the first excitatory transmitter in nerve net neurons, and that GABA acted as an inhibitory signal, possibly released in a refractory manner by the innervated muscle cells themselves.

## 6. Evolution of the Metamera: the invention of gastric pouches

**Figure 6.**
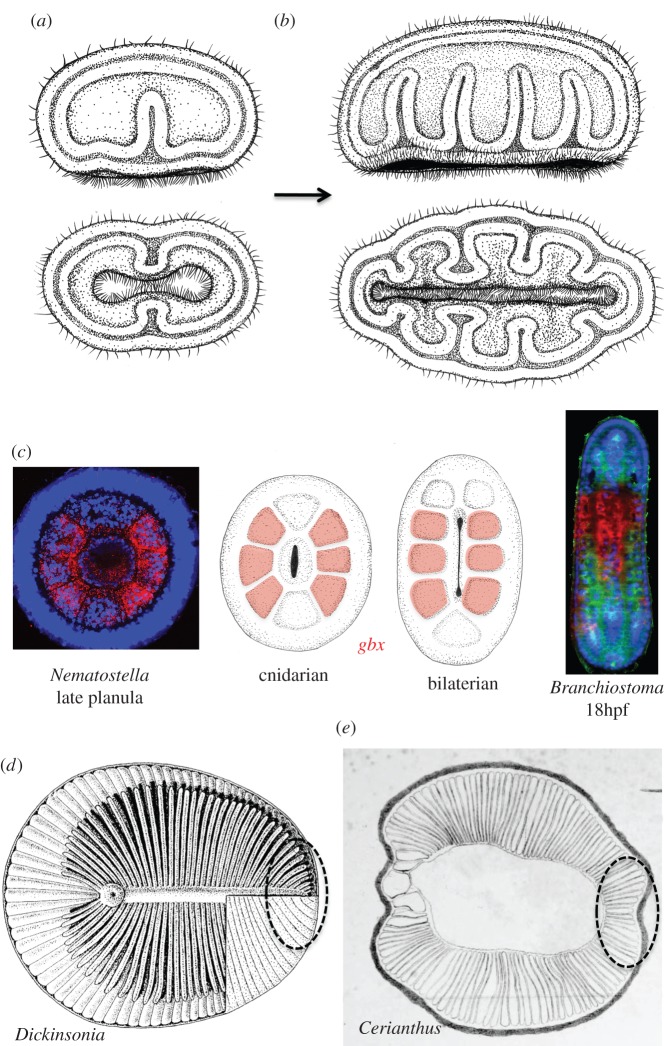
The evolution of gastric pouches. (*a*) Hypothetical modified gastraea with an anterior and a posterior pouch, separated by the primary mesenteries. Establishment of bilateral symmetry. Schematic lateral views (upper panels) and ventral views (lower panels). (*b*) Duplicated pouches leading to two bilateral sets of gastric pouches separated by mesenteries. Schematic lateral views (upper panels) and ventral views (lower panels). (*c*) *Gbx* expression in the gastric pouches of the *Nematostella* late planula (left) and in the amphioxus embryo (right). Midlle panels show *gbx* expression in a general cnidarian versus bilaterian (with slit-like blastopore). (*d*) The metameric gastric diverticula of *Dickinsonia*, adapted with permission from [161]. Stippled circle indicates inferred zone where additional diverticula have been added. (*e*) The metameric gastric pouches of *Cerianthus*, adapted from [162]. Stippled circle indicates growth zone where additional mesenteries develop.

It is reasoned here that one decisive step in the evolution of animals was the formation of a paired fold in the inner lining of the gastraea, the so-called primary mesentery (figure 6*a*). This mesentery then duplicated into two metameric, bilateral series, which enclosed gastric pouches in each half of the body (figure 6*b*). This matches the ground pattern in cnidarians assumed to be ancestral [106]. The obvious advantage of this innovation was the extension of the internal surface, i.e. the surface of the resorptive epithelium, and would have intensified body physiology and probably allowed the maintenance and movement of larger body forms [163] (as were present in the Ediacaran, see below). It has long been suggested that the invention of gastric pouches predated the CBA [164]; this notion can now be revived based on molecular evidence [44]. Beyond that, a plethora of molecular data from sea anemone suggest that bilateral symmetry indeed evolved prior to the cnidarian–bilaterian divergence [165].

### (a) Optimizing water flow

In anthozoan cnidarians, the first manifestation of bilateral symmetry is the formation of a slit-like oral opening, with ciliated grooves on either side that establish water flow [166]. Within the gastric cavity, the mesenteries channel circulation into the pouches. In scyphozoan medusae, such as the moon jellyfish *Aurelia*, the gastric circulation is likewise channeled by the mesenteries (called gastric septa in medusa) into equal streams that pass through the gastric pouches [165].

In contrast with anthozoan cnidarians, the gastric opening of the CBA was most probably facing the substrate, grazing the algal mats with a mucociliary sole (see above). If so, some additional advantages of the newly acquired bilateral symmetry were obvious. Plausibly, body movement changed from multidirectional to unidirectional towards the new anterior end. This would have allowed more targeted food search and avoided passing through areas that had already been grazed (cf. figure 4*a*). Also, a slit-like blastopore would have allowed defecation of the digested and nutrient-deprived mucus with larger non-digestible particles at the new rear end. This is a very straightforward way to evolve the first forward movement: to leave the mucus behind. Apart from that, we can assume that the overall establishment of internal currents passing through the surface-extended pouches might have closely resembled that of some extant cnidarians.

### (b) The keystone: homology of gastric pouches and coelomic pouches

Key to our hypothesis is the presumed homology of cnidarian gastric pouches with the bilaterian coelomic cavities [164]. As outlined elsewhere in more detail [44], new molecular evidence lends strong support to this scenario. Most striking is the spatially restricted expression of *gbx* genes in all but the first pair of pouches, as similarly observed in sea anemone and amphioxus (figure 6*c*). Remarkably, these first pouches give rise to a reduced set of musculature in both sea anemone [106] and amphioxus [167]. Moreover, cnidarian gastric pouches and bilaterian somites share the staggered and collinear expression of *hox* genes with expression boundaries coinciding with boundaries between pouches/somites [44]; and finally, cnidarian gastric pouches express genes that specify somitic musculature in bilaterians, such as *mox* and *twist* [168].

### (c) The *Dickinsoniids*

It is tempting to speculate that these bilateral pouches probably corresponded to the ‘digestive diverticulae’ present in Ediacaran fossils such as *Dickinsonia* [169] (figure 6*d*)*.* These diverticulae have been compared with cnidarian pouches repeatedly [169,170] (figure 6*e*), which would imply that modern cnidarians resemble ‘a grade of Late Precambrian organization from which bilaterians evolved’ [171]. Among the many interpretations that Ediacaran fossils have been subjected to (e.g. [4,172] for an overview), the comparative approach conducted here may thus help to finally select the most plausible variant and place this fossils right in the centre of metazoan evolution and the bilaterian stem [172]. Strikingly, the Dickinsoniids continuously add new mesenteries and diverticules at their ‘posterior’ end [172]; and in a strikingly similar manner additional mesenteries are added in extant cnidarians such as cerianthids or other anthozoans (stippled circles in figure 6*d*,*e*).

## 7. Towards the Bilateria

**Figure 7.**
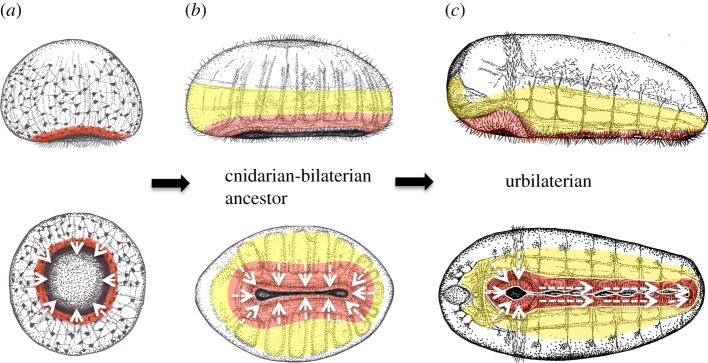
The evolution of the bilaterian nervous system. (*a*) Neuralian ancestor. A nerve net covers the gastrula-shaped animal. Nerve net neurons situated around the digestive opening (red) control feeding. White arrows indicate direction of water flow. (*b*) Cnidarian–bilaterian ancestor. Specialized parts of the nerve net are centred on the slit-like digestive opening: the nerve of the digestive sole (red) and the sensory-integrative centre (yellow), controlling contraction of a bilaterial series of contractile gastric pouches. White arrows indicate direction of water flow. (*c*) Urbilaterian. Motor centre of the ventral nerve cord (red) and sensory-integrative centre (yellow). White arrows indicate direction of water flow.

The last important step in the evolutionary scenario developed here is the median closure of the gastric opening, leaving mouth and anus at opposite ends (figure 7). This step is often recapitulated in bilaterian development, in that both mouth and anal tissue develop from blastoporal tissue (amphistomy) [173] (for alternative views on the interrelationships between gastric opening, mouth and anus, see [44,174]). This closure of the gastric opening along the new ventral midline would have firmly joined together the two bilateral halves of the mucociliary sole. During this transition, specialized subregions of the former nerve net would have given rise to the bilaterian nerve cord. In figure 7, red and yellow demarcate longitudinal subdivisions of nerve cord tissue that derive from different parts of the nerve net: The medial section corresponds to the tissue of the former mucociliary sole; this tissue most probably evolved into the medial motor column of the bilaterian nerve cord [44], and the more lateral section into the lateral sensory-integrative columns. Conserved expression of mediolateral patterning genes supports this scenario, as detailed elsewhere [44]. For example, *nk* homeobox genes genes (*nk2.2* and *nk6*) specify the medial motor centre, wherease *pax* paired-type homeobox genes (*pax37, pax258*) and *msx* specify the more lateral tissue (figure 7).

### (a) From digestive sole to creeping sole and Reissner's fibre

The proposed closure of the slit-like opening of the gastric cavity necessarily impacted on mucociliary feeding, as trapped particles could no longer be internalized alongside the entire gastric slit, but only through the new mouth (and, presumably) anus. In consequence, mucus is no longer transported towards the new ventral midline but towards mouth and anus (white arrows in figure 7*b*,*c*, lower panels). Given that the mucus sticks to the substrate, the anterior-to-posterior movement of the mucus on the ventral body side would have pushed the animals forward. In line with this, creeping forward by means of a mucociliary sole is a prevalent mode of locomotion in many bilaterian phyla. Strikingly, an anterior-to-posterior movement of a mucoid thread propagated by motile cilia, the so-called Reissner's fibre, is observed until today in virtually all chordates (vertebrates and amphioxus) [175]. Our scenario explains the evolutionary origin of this fibre, yet cannot explain why it is so persistent in extant vertebrates.

## 8. Conclusion

The evolutionary scenario developed here sheds new light on the evolution of animal nervous systems, highlighting and postulating two major morphological transitions that increased the efficiency of feeding and locomotion and allowed larger body sizes. First, external digestion with the mucociliary sole would have enabled external digestion and thus enhanced the concentration of nutrients in the gastric cavity. In these animals, a first net of interconnected sensory neurons and contractile effector cells may have evolved that sped up and steered grazing on algal mats. Second, the substantial increase of the internal nutrient-resorbing surface via folding into gastric pouches might have boosted physiology and, consequently, energy supply for locomotion, thus triggering the regionalization and specialization of parts of the nerve net needed for the control of more sophisticated and targeted movements. This ultimately led to convergent centralization events in ctenophores, cnidarians and bilaterians. Necessarily, in the absence of comparative molecular data for most of the cell types discussed here, the postulated sequence of cell-type diversification events underlying these transitions remains tentative. To change this, it is the aim of this review to open up and encourage a new phase of evo-devo, which looks at animal evolution from a cell-type perspective, and to combine these new data with classical comparative anatomy, developmental biology and palaeontology. It is expected that the rapid progress in the profiling and functional characterization of cell types in different animals will boost these efforts.
